# Systematic Experimental Investigation into the Determination of Micromechanical Properties of Hardened Cement Paste Using Nanoindentation—Opportunities and Limitations

**DOI:** 10.3390/ma16041420

**Published:** 2023-02-08

**Authors:** Kristina Raupach, Andreas Bogner, Michael Vogel, Engin Kotan, Frank Dehn

**Affiliations:** 1 Karlsruhe Institute of Technology (KIT), Institute of Concrete Structures and Building Materials (IMB), Materials Testing and Research Institute, Gotthard-Franz-Straße 3, 76131 Karlsruhe, Germany; 2 Kempen Krause Ingenieure GmbH, Ritterstraße 20, 52072 Aachen, Germany

**Keywords:** nanoindentation, heterogeneous material, cement paste, indentation hardness, elastic modulus

## Abstract

The nanoindentation technique is already widely applied in the mechanical characterization of the microstructure of thin films with respect to different materials. Generally, by means of nanoindentation, the hardness and the elastic modulus of materials can be determined with high precision. The focus of these analyses is usually on the materials from the metal, ceramic, and plastics processing industry. The application of nanoindentation in construction science, especially in concrete technology, is a relatively new field of investigation. This study deals with the basic application of nanoindentation for the mechanical characterization of hardened cement paste. In particular, the effects of sample preparation and the selection of the nanoindentation measurement parameters on the obtained results are the main subjects of this investigation. The results re intended to show the opportunities and limitations of analyzing a heterogeneous material such as hardened cement paste. The findings are used to assess the suitability of the nanoindentation method for investigating durability-related damage (e.g., due to freeze–thaw or alkali–silica reaction) in concrete.

## 1. Introduction

Nanoindentation, as a technique used to determine the spatially resolved nano- and micromechanical properties of a material, has gained increasing importance in the field of materials science related to cement and concrete technology (e.g., [1]). However, indenting in highly heterogeneous materials, such as hardened cement paste or even mortar/concrete, leads to challenges concerning the statistical interpretation, the choice of measurement settings, and the exact surface preparation of the specimen.

Concerning cement and concrete technology, nanoindentation is mostly used as a tool to obtain the nano- and micromechanical properties of individual phases and the volume fractions of the unaltered/undamaged binder. Most studies have focused on the nano- and micromechanical properties of cement paste. Here, of great importance are the nano- and micromechanical properties of the calcium silicate hydrate (CSH) phases, which are mostly responsible for the strength of cement paste on the macro scale. From appropriate nanoindentation tests on cement pastes, it is possible to distinguish between low- and high-density CSH [2]. Synthesized CSH was also measured with nanoindentation, and it was concluded that the properties are similar to those of CSH in cementitious composites (e.g., [3]). Furthermore, the creep of hydrated phases of cement pastes during nanoindentation tests was examined [4].

In addition, cement paste blended with different materials, such fly ash (e.g., [5]), slag (e.g., [5]), silica fume (e.g., [6]), nanosilica (e.g., [7]), and polymer-coated silica particles (e.g., [8]), as well as alkali-activated binder systems, were studied (e.g., [9]).

Crucial for all nanoindentation studies are specimens with surfaces as smooth as possible. However, studies that dealt exclusively with how to obtain reproducible and accurate nano- and micromechanical properties of cement pastes are scarce. Miller et al. [10] studied the impact of surface roughness on nanoindentation on cement paste and tried to find a criterion to describe a tolerable level of surface roughness for nanoindentation. Further investigations focused on adjusting the grid size to the material to obtain valuable nanoindentation results [2]. By comparing the following studies [10,11,12,13], there is no unified procedure for the exact surface preparation of specimens and the set parameters such as grid size (225 to 400 indents), spacing between indents (5 to 20 μm), etc.

Therefore, this paper gives a general overview of the factors influencing the quality of nano- and micromechanical properties (indentation hardness and elastic modulus) of hardened cement paste obtained by nanoindentation. The effects of sample preparation and several measurement parameters on the results were exemplarily studied for one nanoindentation setup and three different hardened cement pastes.

Finally, in the Further Research section, the potential of the nanoindentation method for investigating interfacial transition zones (ITZs) and durability-related damages is discussed with focus on frost attack. The results and discussions presented in this paper provide the basis for further research on questions regarding the durability of cementitious materials.

## 2. Materials and Methods

### 2.1. Sample Manufacturing and Surface Preparation

Three different types of cement were used: white (CEM I 42.5 R) and grey (CEM I 42.5 R NA/LA) ordinary Portland cement, as well as grey ordinary Portland cement (CEM I 32.5), according to EN 197-1 [14]. Cylindrical specimens with 10 mm diameter and 20 mm height with a water–cement ratio of 0.40 were prepared.

After the manufacturing process, the specimens were cut along their longitudinal axis and embedded in epoxy resin. White (CEM I 42.5 R) and grey (CEM I 32.5) ordinary Portland cement paste specimens were cut approximately at the age of 28 days and grey ordinary Portland cement paste specimens (CEM I 42.5 R NA/LA) at the age of about 3 years. The used precision sectioning saw was an IsoMet 1000 from Buehler (Buehler Ltd., ITW Test & Measurement GmbH, Leinfelden-Echterdingen, Germany). For the embedding process, a Cast N’ Vac 1000 Vacuum System from Buehler was used. The embedding forms were cylindrical and had a diameter of 32 mm. The used epoxy resin system was Epoxy 2000 resin and hardener from Cloeren Technology GmbH (Wegberg, Germany) (mix ratio 100:48). Blue coloring from Buehler was added to highlight the contrast between the specimen and epoxy resin. During the embedding process, a vacuum was twice created and drawn to ensure the complete filling of pores and cracks. The cross-section surface of the embedded specimen was then ground and polished. For error-free nanoindentation tests, a smooth and plain specimen surface is required [15].

A standardized grinding and polishing procedure to prepare cement paste surfaces for nanoindentation was previously unavailable. In the literature, various different procedures have been used (e.g., [2,10,16]). The herein used grinding and polishing procedure consisted of four steps. An automatic grinding and polishing machine (AutoMet/EcoMet 250 from Buehler) was used [17]. First, the specimen surface was ground with a 45 μm Apex DGD diamond grinding disc (Buehler) for three minutes. A compressive force of 22 N and a rotation speed of 300 rpm were applied. The disc and the sample holder moved in opposite directions. The polishing procedure was followed with a MetaDi diamond suspension of 9, 3, and 1 μm on TriDent cloth (all from Buehler) for four minutes each. A compressive force of 13 N and a rotation speed of 150 rpm were applied. The disc and sample holder moved in the same direction. Finally, the specimen surface was polished with 0.05 μm MasterPrep aluminum oxide suspension on a Texmet cloth (both from Buehler) for two minutes. A compressive force of 18 N and a rotation speed of 150 rpm were used. The disc and the sample holder moved in the same direction. In between the single steps, the surface was cleaned with dishwashing liquid in an ultrasonic bath for three minutes.

**Figure 1:**
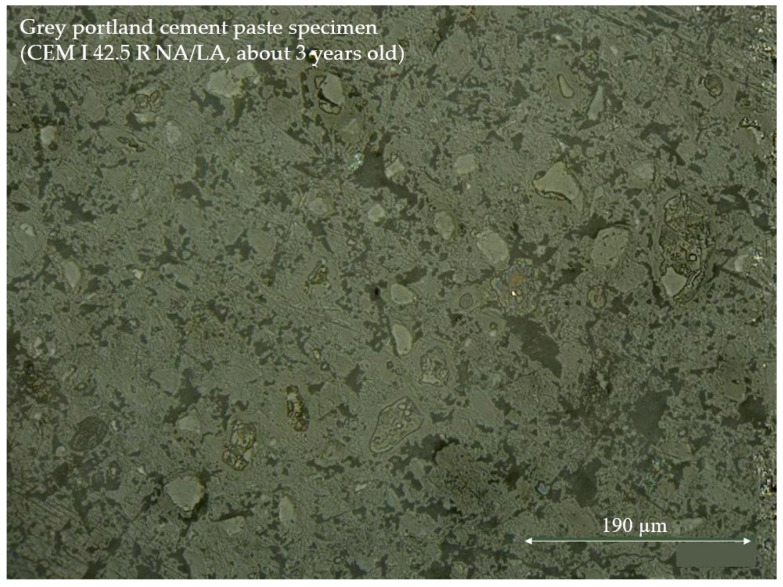
Fully ground and polished cross section-surface (example).

To characterize the fully ground and polished cross-section surfaces of the cement paste specimens and to ensure the replicability of the grinding and polishing procedure, the fully ground and polished cross-section surfaces were investigated under a digital microscope (VHX-2000 series, Keyence Deutschland GmbH, Neu-Isenburg, Germany). A picture of the fully ground and polished cross section-surface of a grey ordinary Portland cement paste specimen (CEM I 42.5 R NA/LA, about 3 years old), on which the nanoindentation tests for the investigation of the statistical analysis and the choice of settings were performed, is shown in Figure 1.

### 2.2. Nanoindentation on Cement Paste

Nanoindentation tests on the manufactured and prepared cement paste specimens were performed with an Ultra Nanoindentation Tester UNHT^3^ (Anton Paar GmbH, Graz, Austria; load range: 0.01 mN to 100 mN, penetration depth range up to 100 μm). The measuring head consisted of an indenter and an additional sensor called the reference, which was used to detect the distance between the resting position of the indenter tip and the specimen surface. For nanoindentation on cement paste, a common Berkovich indenter was used. Before each indentation test, a procedure to adjust the depth offset (ADO) was carried out in the form of a quick single indentation to set up the depth sensor measurement range for the specific position of the sample for the following measurements.

Prior to an indentation test, the parameters for the ADO, the measurement, the sensor ranges, as well as the measurement mode needed to be set. The parameters used in this study are listed in Table 1. In general, the parameters need to be chosen in correspondence with the surface roughness of the specimen. A detailed description of each parameter can be found in [18].

Concerning the measurement mode, Anton Paar Indentation Software allowed us to choose between the Quick, Simple, and Advanced Matrix Modes. All modes allowed us to perform several single indentation tests with the same measurement parameters, arranged with constant distances in a measurement grid. As cement paste is a highly heterogeneous material, such grid nanoindentation tests need to be applied to characterize the nano- and micromechanical properties of cement paste [12].

Selecting the Quick Matrix Mode, the reference remains at the surface of the specimen throughout the whole grid indentation test, whereas the indenter is raised and once again lowered after each single indentation using Simple or Advanced Matrix Mode. Thereafter, setting the Quick Matrix Mode can significantly reduce the duration of the grid indentation test compared with Simple and Advanced Matrix Modes. The Advanced Matrix Mode allows, in comparison with the Simple and Quick Matrix Modes, more flexibility in locating the single indentations and setting individual measurement settings for each single indentation. The Simple and Quick Matrix Mode only allow rectangular positions of indents with constant spacing and equal parameters for all indentations [18].

**Figure 2:**
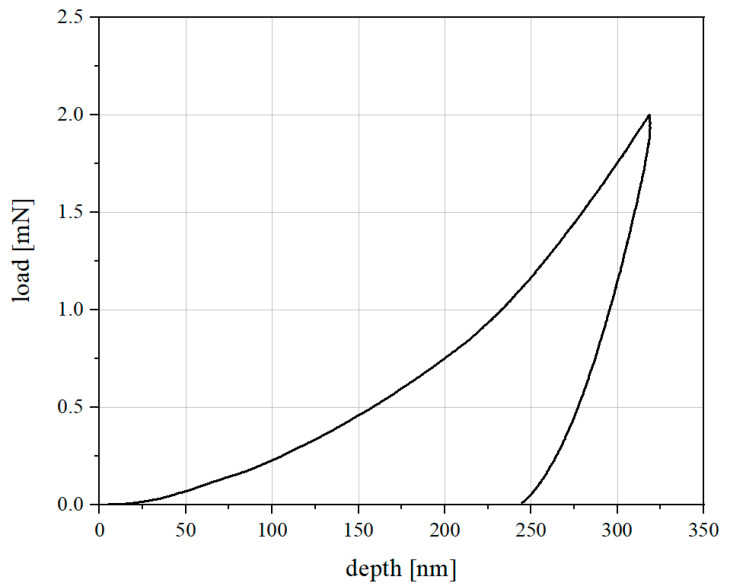
Typical load–depth curve due to a single indentation test.

Each single indenation test results in a load-depth curve as examplarily shown in Figure 2.

From each load–depth curve, the mechanical parameters, indentation hardness and elastic modulus, commonly used for characterization of the micro- and nanomechanical properties of cement paste were determined following the commonly used Oliver and Pharr analysis method [19,20] and a Poisson’s ratio of 0.2 [12,13].

## 3. Results and Discussion

### 3.1. Statistical Analysis

The measured values for indentation hardness and elastic modulus of three 20 × 20 grid indentations carried out on three different positions of a sample of hardened cement paste (CEM I 42.5 R NA/LA, about 3 years old) showed heavy scattering, which was expected due to the high heterogeneity of the tested cement pastes. The parameters were identically set for all indentations and can be found in detail in Section 2.2.

**Figure 3:**
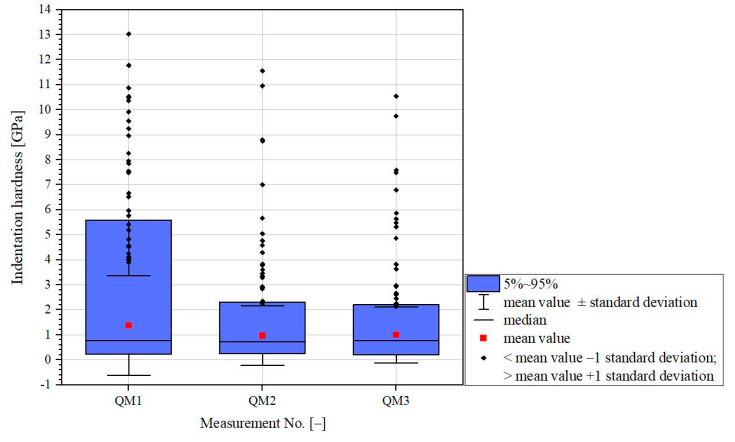
Statistical analysis of indentation hardness of three grid indentations (QM1 to QM3) on three positions of a sample of hardened cement paste (CEM I 42.5 R NA/LA, about 3 years old) (example).

A statistical analysis considering all 400 parameters was unfeasible. The standard deviation of all three grid indentations was greater than the mean value (see Figure 3).

**Figure 4:**
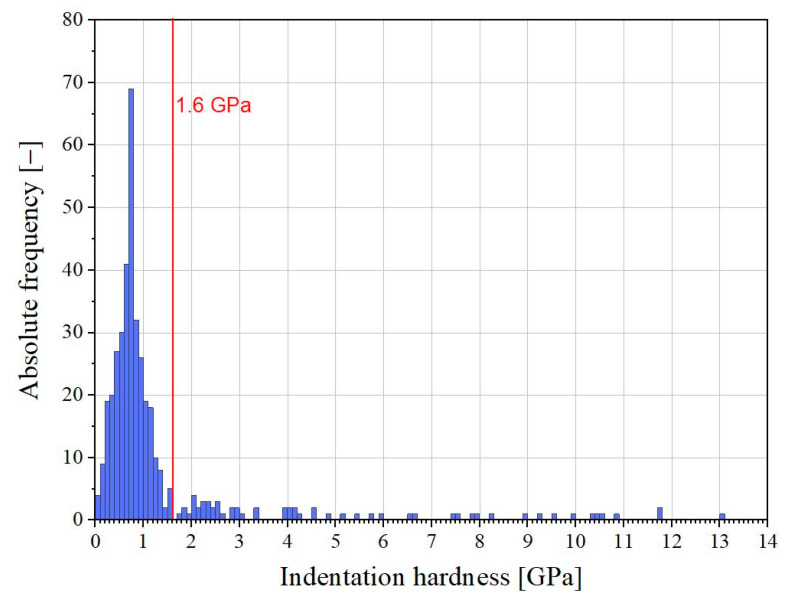
Absolute frequency of indentation hardness (example).

To determine the mean nano- and micromechanical properties of the tested cement pastes, the measured values of indentation hardness greater than 1.6 GPa and of elastic modulus greater than 55 GPa were treated as outliers and therefore excluded from the statistical analysis (see Figure 4). As a criterion for the limit values, the mean value multiplied by two was used. The distribution of the measured values of indentation hardness and modulus thereafter nearly corresponded to a statistical normal distribution (Gaussian distribution, see Figure 4), and the mean indentation hardness and mean elastic modulus of cement paste could be determined.

**Figure 5:**
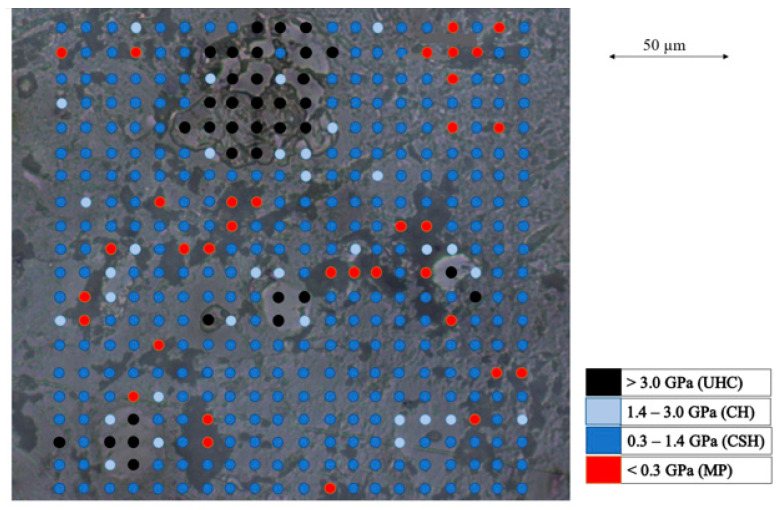
Surface map of indentation hardness (example).

The application of the surface mapping method in combination with measured values of indentation hardness and elastic modulus for a predominant indentation in a specific cement paste component (unhydrated cement clinker (UHC), calcium hydroxide (CH), calcium silicate hydrate (CSH), and micropores (MPs)) found in the literature (based on [11,21]) allowed us to assign the indentation hardness and elastic modulus values to different phases. An example of a surface map of the indentation hardness is shown in Figure 5.

### 3.2. Measurement Settings

To obtain reproducible nanoindentation results, the impact of varying loads (2 to 100 mN), grid sizes (5 × 5, 10 × 10, and 20 × 20), spacing between indents (5, 10, and 20 μm), and measurement modes (Quick, Simple, and Advanced Matrix) was investigated “on a sample of hardened cement paste (CEM I 42.5 R NA/LA, about 3 years old)”. All other parameters (adjust depth offset (ADO), measurement preferences, and sensor ranges) were kept constant throughout all nanoindentation tests. The set parameters for the ADO, measurement preferences, and sensor ranges can be found in Section 2.2.

#### 3.2.1. Maximum Load

**Figure 6:**
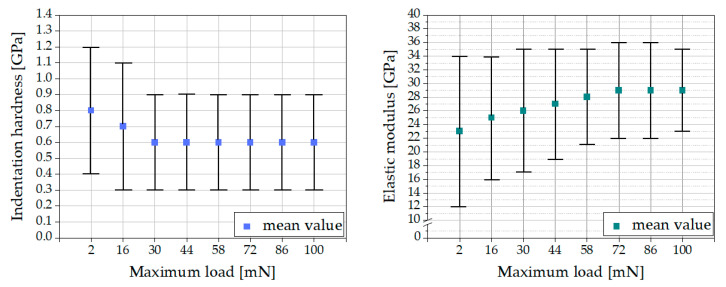
Impact of maximum load on indentation hardness (**left**) and elastic modulus (**right**).

In the literature a maximum load of 2 mN is usually set for nanoindentation tests on cement paste (e.g., [10,11,12]). As our research goal was to determine the mean mechanical properties of cement paste rather than to investigate the mechanical properties of the single components, we investigated whether a maximum load of 2 mN would also be appropriate for this purpose. Therefore, the maximum load was varied between 2 and 100 mN, because 100 mN was the maximum load possible for the UNHT^3^ Nanoindenter. The results are shown in Figure 6.

The indentation hardness decreased with increasing maximum load, whereas the elastic modulus increased with increasing maximum load, but the standard deviations of all measurements overlapped. Therefore, the impact of maximum load on the results was insignificant for the herein performed nanoindentation tests. To compare the results with those in the literature, the maximum load of 2 mN was applied.

#### 3.2.2. Grid Size

**Figure 7:**
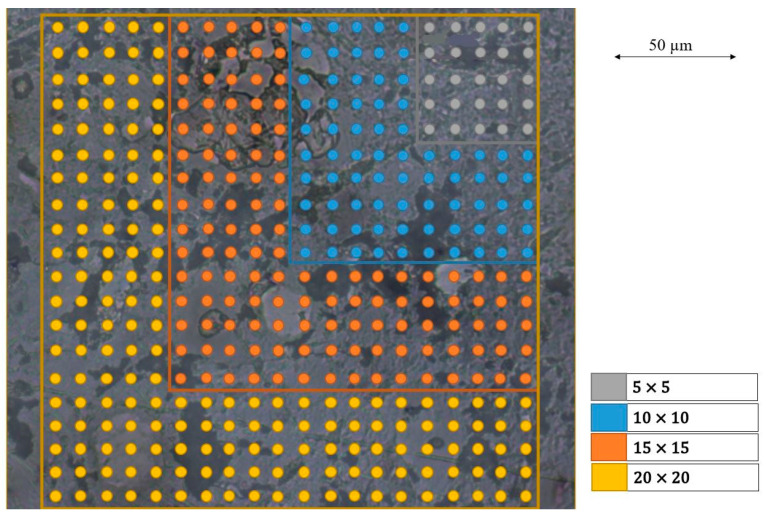
Included nanoindentation results in the statistical analysis.

According to [22], the chosen grid size must be adjusted to the scatter of the nanoindentation results. Anton Paar chose a grid size of 5 × 5 for reference nanoindentation tests on fused silica. The maximum grid size in consideration of the set parameters described in Section 2.2 was 20 × 20 due to the storage capacity of the UNHT^3^ Nanoindenter. The mean indentation hardness and mean elastic modulus calculated from the data of a 20 × 20 grid and from 5 × 5, 10 × 10, and 15 × 15 subgrids, as well as a statistical analysis, are shown in Figure 7.

**Figure 8:**
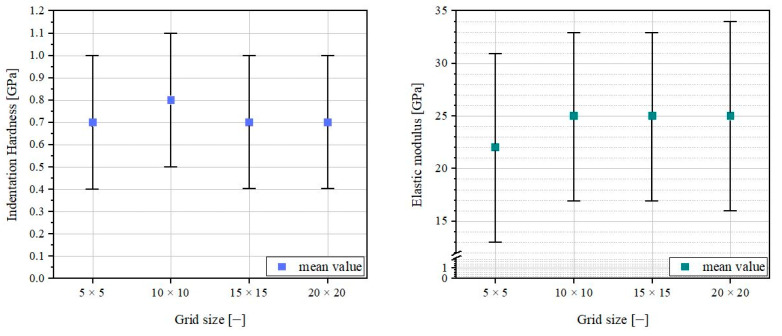
Impact of grid size on indentation hardness (**left**) and elastic modulus (**right**).

The achieved results with the 5 × 5, 10 × 10, 15 × 15, and 20 × 20 grid sizes measurements are depicted in Figure 8.

**Figure 9:**
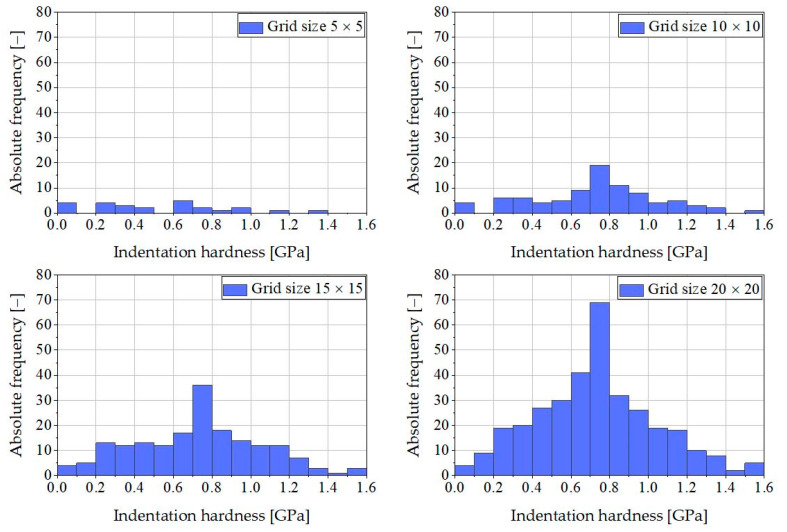
Impact of grid size (5 × 5, 10 × 10, 15 × 15, and 20 × 20) on the absolute frequency of indentation hardness.

The results in Figure 8 seem to be nearly independent of the chosen grid size. The related histograms for the indentation hardness are shown in Figure 9.

The histograms have approximately the shape of a Gaussian distribution when measurement results using grid sizes larger than 10 × 10 are considered in the statistical analysis.

#### 3.2.3. Spacing

**Figure 10:**
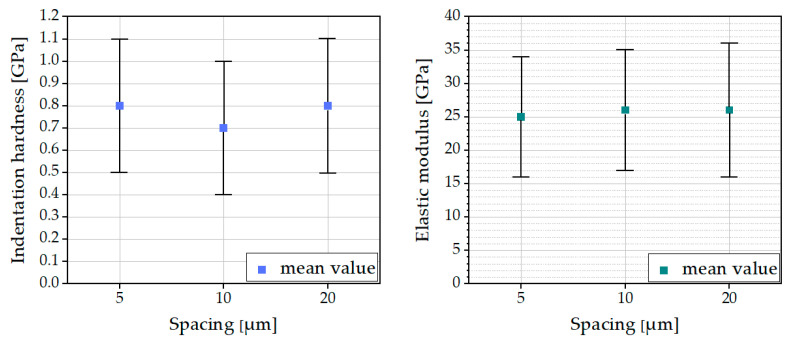
Impact of spacing on indentation hardness (**left side**) and elastic modulus (**right side**).

According to [23] an appropriate choice of the spacing between the indents is important for preventing any mutual influence from adjacent indentation tests on the results. The spacing between indents was varied from 5, 10, to 20 μm. The results are shown in Figure 10.

The results seem to be nearly independent of the chosen spacing. In conjunction with the results of the investigation of the grid size, a grid size of 20 × 20 in combination with a spacing of 10 μm was preferred for our performed tests. An area of 190 × 190 μm nearly equaled the image size of 255 × 202 μm of the UNHT^3^ microscope.

#### 3.2.4. Mode

**Figure 11:**
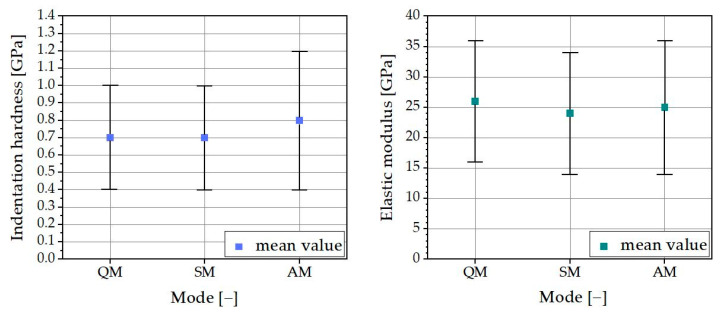
Impact of mode on indentation hardness (**left side**) and elastic modulus (**right side**).

Figure 11 shows the results of a grid indentation test using the Quick Matrix (QM), Simple Matrix (SM), and Advanced Matrix (AM) modes.

**Figure 12:**
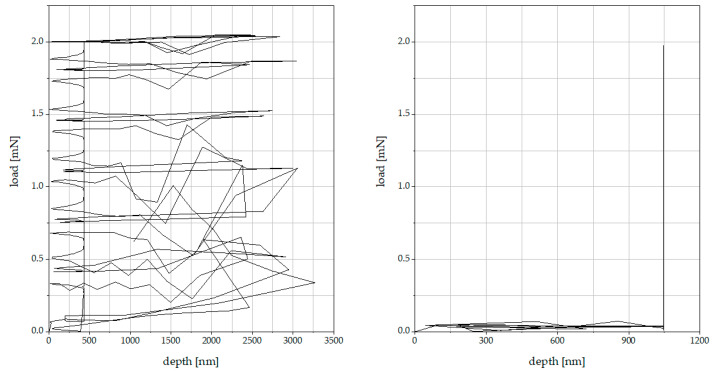
Scattering in recorded load–depth curves (example).

According to the nanoindentation results, all three modes can be used for nanoindentation on cement paste. Nevertheless, it is important to know that when using the Quick Matrix Mode, no errors occurred throughout all the single indentations. All load–depth curves were inconspicuous and could be used to determine the indentation hardness and elastic modulus. Using the Simple and Advanced Matrix mode, up to 21% of the load–depth curves were faulty. Either no load-depth curve was recorded or the recorded load–depth curve showed severe scattering and a calculation of indentation hardness and modulus was unfeasible (see Figure 12).

The faulty load–depth curves were attributable to the high heterogeneity of the cement paste in combination with the Anton Paar referencing system, which were more significant when using the Simple and Advanced Matrix modes than when in Quick Matrix mode. The choice of mode depends on the surface roughness of each specimen. When using Quick Matrix mode, the surface should be as smooth as possible to reduce the risk of damaging the reference while it remains at the specimen surface throughout the whole measurement. If a smooth surface cannot be guaranteed, despite the errors that may occur, Simple or Advanced Matrix mode should be used instead.

### 3.3. Surface Preparation

**Figure 13:**
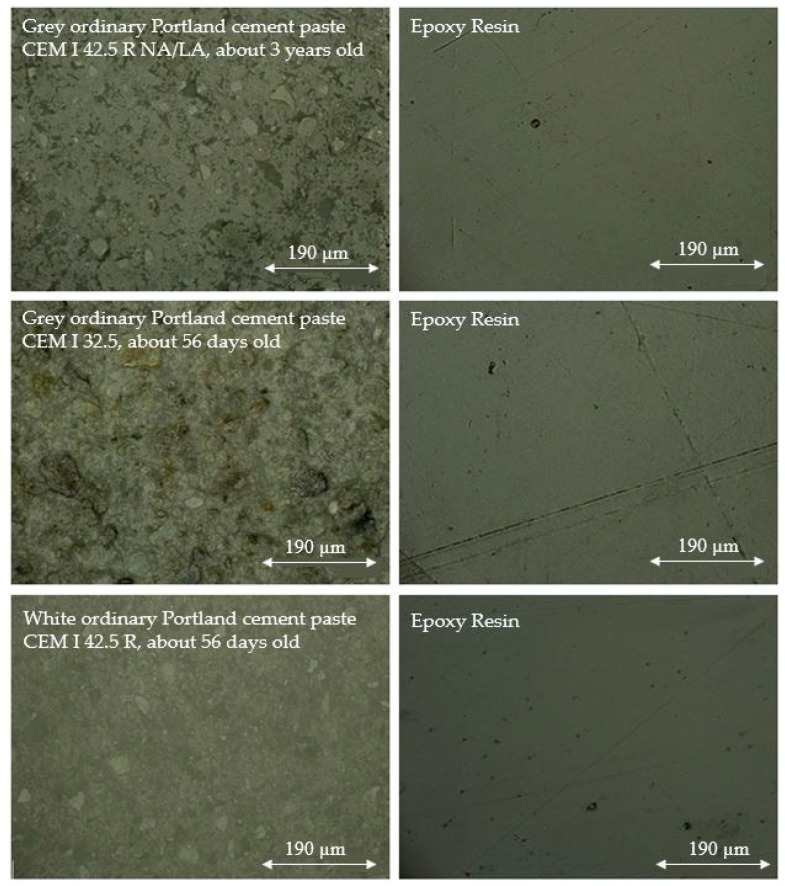
Microscopic images of ground and polished specimen surfaces (example).

The surface preparation procedure explained in Section 2.1 was used to prepare manufactured ordinary Portland cement paste (CEM I 42.5 R NA/LA, about 3 years old) for nanoindentation. For the white ordinary Portland cement paste specimens (CEM I 42.5 R, about 56 days old) and the grey ordinary Portland cement paste specimens (CEM I 32.5, about 56 days old), the grinding and polishing procedure in Section 2.1 was slightly altered due to the heavy wear of the used Apex DGD cloth. Two minutes were added to the grinding procedure with the Apex DGD cloth, and a grinding procedure with SiC paper (P400 and P600) for one minute each was added before polishing the specimen surface with the MetaDi diamond suspension (9 μm). Microscope images of the surfaces of the three different cement paste specimens are shown in Figure 13.

The surfaces of the epoxy resin of the grey (CEM I 42.5 R NA/LA) and white (CEM I 42.5 R) ordinary Portland cement paste resembled one another, whereas the appearance of the epoxy resin of the grey ordinary Portland cement paste (CEM I 32.5) showed more grooves. In the case of the grey ordinary Portland cement paste (CEM I 42.5 R NA/LA), the microstructure of the cement paste was most clearly visible. Nanoindentation tests could be performed on the grey ordinary Portland cement paste (CEM I 42.5 R NA/LA) and white ordinary Portland cement paste (CEM I 42.5 R), whereas this was not possible on the grey ordinary Portland cement paste (CEM I 32.5) because the ADO could not be properly performed. Therefore, using the described surface preparation procedure, the highest quality of the surface (microstructure most clearly visible and no pronounced grooves) was obtained for the about three-year-old grey ordinary Portland cement paste (CEM I 42.5 R NA/LA). Unfortunately, it could not be finally resolved whether the cement type and/or the specimen age was responsible for the different qualities of the surfaces or whether this was due to the slight alteration in the polishing procedure. However, one can say that the selected polishing and grinding procedure is of great importance for the performance of nanoindentation tests on cement pastes.

## 4. Summary and Conclusions

The performed nanoindentation tests on cement pastes showed how reproducible and accurate nanoindentation results can be obtained.

A determination of the mean nano- and micromechanical properties of cement pastes is possible by excluding the measured values of indentation hardness greater than 1.6 GPa and those of elastic modulus that are greater than 55 GPa. The excluded values can be mostly assigned to a predominant indentation in unhydrated clinker particles and calcium hydroxide.

With regard to the nanoindentation tests described above, the following settings were found to be suitable: The impact of maximum load (2 to 100 mN) on the results was insignificant for the herein nanoindentation tests performed on cement pastes. To compare the results with those in the literature, the maximum load of 2 mN should be applied.

In addition, the nanoindentation results seemed to be nearly independent of the chosen grid size, and the histograms approximately had the shape of a Gaussian distribution when more than 10 × 10 grid size measurement results were considered in the statistical analysis. In combination with the nearly independent results of the spacing (5, 10, and 20 μm), a grid size of 20 × 20 in combination with a spacing of 10 μm is most suitable for the Ultra Nanoindentation Tester UNHT^3^ produced by Anton Paar. An area of 190 × 190 μm nearly equals the image size of 255 × 202 μm of the UNHT^3^ microscope.

Concerning the choice of measurement mode (Quick, Simple, or Advanced Matrix), for a smooth specimen surface, Quick Matrix is recommended, whereas the Simple or Advanced Matrix should be used if a smooth surface cannot be guaranteed.

The importance of surface preparation on cement pastes can be seen as essential for performing nanoindentation tests. The described procedure was most suitable for three-year-old grey ordinary Portland cement paste. Whether other cement types and specimen ages require modifications of the surface preparation procedure should be further investigated.

## 5. Further Research

In addition to investigating the nano- and micromechanical properties of the individual phases and their volume fractions in the binder, interfacial transition zones can also be studied with nanoindentation. This takes advantage of the high spatial resolution of the method, which makes it possible to specifically investigate the individual mechanical properties of individual zones of a sample that are not accessible with macroscopic investigations. There are, for example, studies of the ITZ between cement paste and aggregates in concrete (e.g., [24]), between cement paste and different types of fibers (e.g., [21]), and even of new and old ITZs in recycled aggregated concrete [25].

The ability to probe the mechanical properties of the ITZs, which are most susceptible to damage, as well as to investigate deteriorations spatially resolved along a damage gradient, opens up interesting opportunities to study durability-related damages. There are some studies of cement paste or concrete exposed to durability-relevant stresses such as leaching (e.g., [26]), sulfate attack (e.g., [27]), alkali–silica reaction (e.g., [28]), carbonation (e.g., [11]), and high temperatures [29]. However, there are only a few publications in each of these areas. As an example, the potential of nanoindentation for investigating frost attack is briefly discussed below.

With regard to durability-related damage modeling or probabilistic lifetime prediction, it is important to have a clearly defined damage or boundary condition. For example, in the case of frost attack (without deicing agent), the limit state is reached when the actual degree of saturation in the concrete pore system at a point in time reaches the critical degree of saturation [30]. If this limit state has been reached, only a few freeze–thaw cycles are required to initiate internal structural damage in the concrete [31]. This microstructural damage in the concrete can be determined on a macroscopic level by measuring the decrease in the relative dynamic modulus of elasticity of the concrete. At the moment when the measured relative dynamic modulus of elasticity of the concrete decreases, the internal damage is accompanied by significant microcracking in connection with increasing porosity of the stressed hardened cement paste.

In addition to the assessment of the durability-related behavior of concrete based on experimental ultrasonic measurements, the use of a multiscale ultrasonic pulse velocity model for multiphase concrete materials is recommended [32]. By means of the homogenization approach, the elastic parameters and ultrasonic pulse velocity of cement paste, mortar, and concrete can be predicted considering the hydration process. Here, a promising approach is the implementation of the results of nanoindentation of hardened cement paste into the mentioned homogenization method. This allows a first correlation of the results of the ultrasonic measurement with the results of the nanoindentation.

However, there is still discussion about when frost-induced micro cracking inside hardened cement paste or in the interfacial transition zone (ITZ) between aggregate and hardened cement paste actually occurs. In relation to the durability-relevant exposure of concrete, it is important to use sophisticated measurement methods at the microstructure level to make reliable statements about the behavior of the material at the macrostructure level. Nanoindentation has been proven to be a helpful method in this case, as it was possible to determine the change in microporosity within hardened cement paste as well as within the ITZ between hardened cement paste and steel fibers of frost-stressed ultra-high-performance concrete [25].

Based on continuous multiscale micromechanics, the ice-induced influences on the elastic properties of mortar were simulated. In addition, using the rigid-body spring method (RBSM), the nonlinear compressive, uniaxial tensile, and splitting behaviors were predicted [33]. Here, in particular, the frost-related microcracking of hardened cement paste and ITZ could be identified. Therefore, the possibility arises to compare the results of the nanoindentation with the described simulation results.

However, depending on the material used, accurate specimen preparation is extremely important in order to be able to clearly distinguish between system-related (e.g., sawing, grinding, and polishing of the specimen) and test-related (e.g., freeze–thaw application) results.

Nevertheless, microstructural investigation by means of nanoindentation of normal-strength concretes exposed to durability-relevant stresses is of great interest in order to be able to describe the damage processes occurring at the macrostructural level. There are still many open questions in this field, which can be successfully answered in the future with the proper and professional application of nanoindentation.

## Figures and Tables

**Table 1:** Set parameters for nanoindentation.

**Adjust Depth Offset Parameters (ADO)**	
Approach speed	25,000 nm/min *
Contact force	0.05 mN
Characterization force	0.50 mN
Contact stiffness threshold	150.0 μN/μm
Preapproach (indenter)	10%
Preapproach (reference)	40%
Contact force (reference)	0.50 mN
**Measurement preferences**	
Indenter approach parameters	
Approach speed	25,000 nm/min *
Approach distance	2000 nm
Contact stiffness threshold	150 μN/μm
Contact load	0.02 mN
Reference approach parameters	
Approach speed	60,000 nm/min *
Contact load	0.02 mN
Preapproach	40%
Reference–indenter autotuning	Active
**Sensor ranges**	
Reference load range	20 mN
Indenter load range	100 mN
Depth range	10 μm

* Notification: The approach speed parameters highly depend on the surface roughness of the specimen. For further investigations, these parameters should be reduced to avoid indenter and reference damage.

## Data Availability

Not applicable.
